# Engineered Dynamic Hydrogel Niches for the Regulation of Redox Homeostasis in Osteoporosis and Degenerative Endocrine Diseases

**DOI:** 10.3390/gels10010031

**Published:** 2023-12-30

**Authors:** Weihao Yuan, Jiankun Xu, Na Yang, Han Wang, Jinteng Li, Mengyao Zhang, Meiling Zhu

**Affiliations:** 1 The Eighth Affiliated Hospital, Sun Yat-Sen University, Shenzhen 518033, China; yangn68@mail2.sysu.edu.cn (N.Y.);; 2 Weintraub Center for Reconstructive Biotechnology, Division of Advanced Prosthodontics, School of Dentistry, University of California, Los Angeles, CA 90095, USA; 3 Musculoskeletal Research Laboratory, Department of Orthopedics & Traumatology, Li Ka Shing Institute of Health Sciences, The Chinese University of Hong Kong, Hong Kong 999077, China

**Keywords:** osteoporosis, degenerative endocrine disease, engineered hydrogel niches, redox homeostasis

## Abstract

Osteoporosis and degenerative endocrine diseases are some of the major causes of disability in the elderly. The feedback loop in the endocrine system works to control the release of hormones and maintain the homeostasis of metabolism, thereby regulating the function of target organs. The breakdown of this feedback loop results in various endocrine and metabolic disorders, such as osteoporosis, type II diabetes, hyperlipidemia, etc. The direct regulation of redox homeostasis is one of the most attractive strategies to redress the imbalance of the feedback loop. The biophysical regulation of redox homeostasis can be achieved through engineered dynamic hydrogel niches, with which cellular mechanics and redox homeostasis are intrinsically connected. Mechanotransduction-dependent redox signaling is initiated by cell surface protein assemblies, cadherins for cell–cell junctions, and integrins for cell–ECM interactions. In this review, we focused on the biophysical regulation of redox homeostasis via the tunable cell–ECM interactions in the engineered dynamic hydrogel niches. We elucidate processes from the rational design of the hydrogel matrix to the mechano-signaling initiation and then to the redox response of the encapsulated cells. We also gave a comprehensive summary of the current biomedical applications of this strategy in several degenerative endocrine disease models.

## 1. Introduction

Endocrine diseases, encompassing a broad spectrum of conditions affecting hormonal regulation, pose significant challenges to human health and quality of life. These diseases, especially their degenerative subtypes like diabetes, thyroid disorders, and osteoporosis, have become increasingly prevalent and complex in aging populations worldwide [1,2]. Such conditions not only lead to a decline in life quality but also place a substantial burden on healthcare systems. In the realm of tissue engineering and regenerative medicine, scaffolds derived from biomaterials have emerged as a beacon of hope [3,4,5,6]. Treatments like bisphosphonates, calcitonin, hormone replacement for osteoporosis, insulin therapy and oral hypoglycemics for diabetes, and hormone replacement for thyroid disorders, while effective, have drawbacks such as side effects, the need for careful dose management, and concerns about long-term efficacy. These challenges underscore the need for innovative approaches like dynamic hydrogels, offering more tailored and potentially less invasive options.

Hydrogels, in particular, stand out as ideal mediums for both drug and stem cell-based therapies. These are extensively hydrated polymer constructs, and their design and characteristics can be customized to meet specific requirements [7,8,9,10,11]. Prior research indicates that various hydrogels, such as polysaccharides [12,13,14], polyacids [15,16], polymethacrylates [17,18], fibrous hydrogels [19,20,21], and other self-assembled hydrogels [22,23,24], can be designed to mimic the structure of the absent extracellular matrix (ECM) in tissue injuries, thereby promoting internal cell growth and differentiation. Dynamic network hydrogels represent an advanced subset of these materials. They are characterized by their ability to adapt and change in response to environmental stimuli, making them particularly effective for regenerative applications. These hydrogels offer enhanced support for the growth and differentiation as well as the attraction of immunoregulatory cells compared to those containing only stem cells. As such, these show promise as tools for early detection and treatment of degenerative endocrine diseases, potentially reducing the onset and severity of symptoms and complications. Dynamic hydrogels thus show great promise as tools for the early detection and treatment of these diseases, potentially reducing the onset and severity of symptoms and complications. While there have been significant advancements in the creation of dynamic hydrogels over the years, there is still much to uncover regarding their optimal design and their medical applications, especially in the context of maintaining redox balance in degenerative endocrine diseases.

Herein, we introduce and illustrate in detail the design principles of dynamic hydrogels with respect to how they can be finely tailored towards the regulation of redox homeostasis through biophysical cell–material interactions and the biomedical applications of dynamic hydrogels in degenerative endocrine diseases. We hope this review can benefit both biomaterial researchers and endocrinologists in further studying the mechanisms of disease and expanding their work’s effectiveness.

## 2. The Design of Dynamic Hydrogels

### 2.1. Degradation-Reliant Dynamic Hydrogels

Degradation-reliant dynamic hydrogels, known for their robust yet selectively degradable crosslinks, represent a significant advancement in biomaterial science. These hydrogels are engineered to maintain structural integrity while allowing controlled degradation through specific mechanisms like hydrolytic breakdown, enzymatic degradation, and light-responsive degradation [25,26,27,28,29,30,31,32,33,34,35,36]. This selective degradability is crucial for applications in tissue engineering and drug delivery, where the temporal and spatial control of material properties is essential.

**Figure 1:**
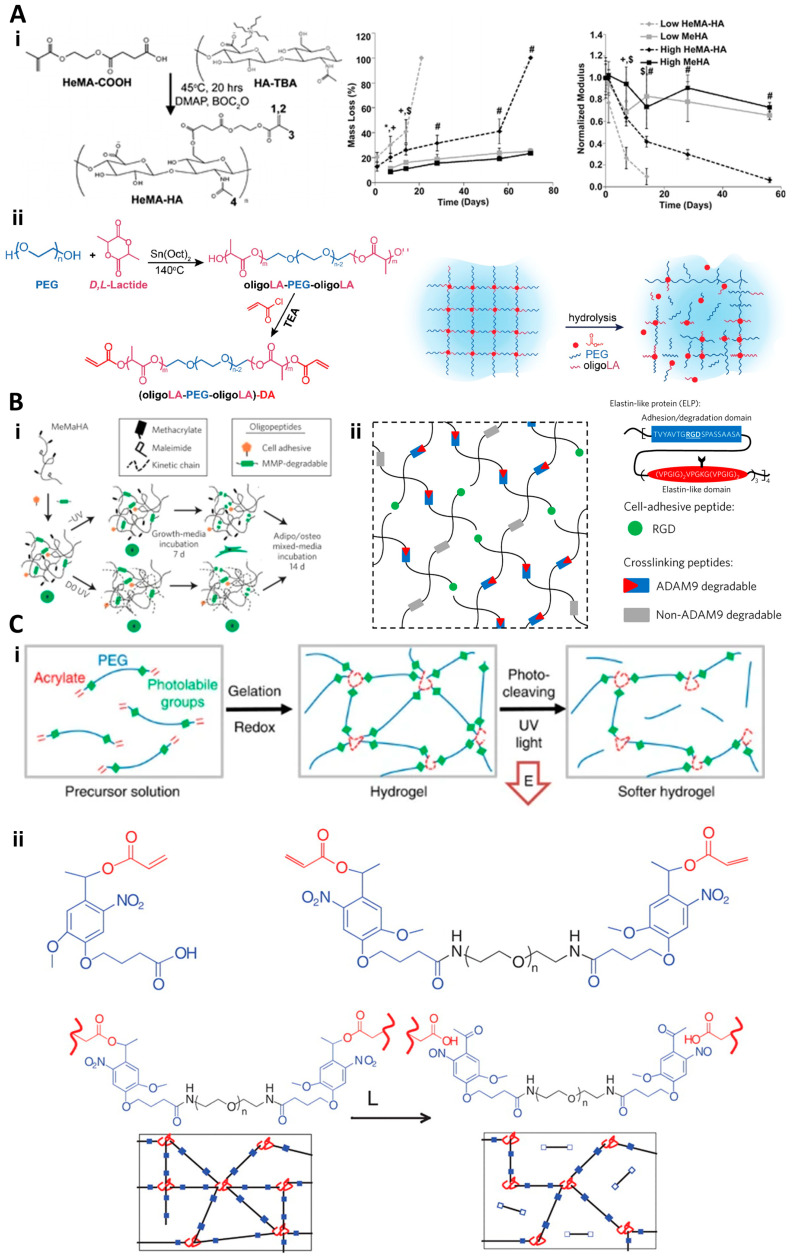
(**A**) (**i**) Creation of HeMA-HA hydrolytically degradable hydrogels [37]. #, +, $, *: significant difference, *p* < 0.05. (**ii**) Formation and hydrolytic breakdown diagram of PEG-oriented hydrolytically degradable hydrogels [38]. (**B**) (**i**) Development and cell-induced enzymatic breakdown of HA hydrogels [39]. (**ii**) Composition of PEG hydrogels with MMP-sensitive ELP-RGD peptide links. (**C**) (**i**) Illustration of light-induced degradation in PEG-based dynamic hydrogels responsive to light [23]. (**ii**) Chemical make-up and light-triggered mechanism in crosslinkers based on photo-degradable nitrobenzyl [39,40].

For hydrolytically degradable dynamic hydrogels, the breakdown typically involves the hydrolysis of covalent interactions. The rate and extent of this degradation can be finely tuned by altering the crosslinking density, as demonstrated by Burdick and colleagues in their development of hydroxyethyl methacrylate (HEMA)-modified polysaccharide-based hydrogels. These hydrogels showcased the ability to adjust degradation rates, offering the potential to customize the release of therapeutic agents or the gradual exposure of embedded cells to the surrounding environment [37] (Figure 1(Ai)). Ding et al. further expanded on this concept by creating a hydrogel system where the hydrolytic degradation rate could be precisely controlled [38] (Figure 1(Aii)). This adjustability is especially beneficial in regenerative medicine, as it allows for the fine-tuning of the hydrogel environment to optimize cell behavior, such as enhancing the proliferation and differentiation of encapsulated MSCs.

Enzymatically degradable hydrogel designs draw inspiration from natural biomacromolecules. These hydrogels are designed to include enzyme-cleavable peptide sequences, enabling cells to actively participate in remodeling their microenvironment. This feature is particularly useful in replicating natural tissue dynamics, where cellular activities are closely intertwined with the surrounding ECM. Burdick’s group formulated an MMP-sensitive peptide-crosslinked HA hydrogel to study how cell-mediated enzymatic degradation impacts encapsulated cell behavior [39] (Figure 1(Bi)). Heilshorn et al. introduced an enzyme-responsive platform using a composite protein structure integrated with a non-active domain and an active domain with cell-adhesive moieties [23] (Figure 1(Bii)). This design not only supports cell adhesion and proliferation but also allows for the controlled release of bioactive molecules, enhancing tissue regeneration and repair.

Additionally, there is growing interest in light-responsive degradation hydrogels because of their controlled degradation. These hydrogels are designed to degrade upon exposure to specific light wavelengths, providing an unprecedented level of control over the degradation process. Anseth and colleagues designed nitrobenzyl-crosslinked hydrogels that break down when exposed to visible light [39,40] (Figure 1C). When exposed to visible light, these hydrogels degrade in a controlled manner, allowing for the spatially and temporally precise release of encapsulated drugs or growth factors. This capability is particularly advantageous in wound healing and tissue engineering applications, where localized and timed therapeutic interventions are crucial.

All these hydrogel examples exhibit how specific cellular behaviors, such as adhesion, migration, and differentiation, can be directed by adjusting hydrogel monomer structures and scaffold degradation properties. This capability is particularly advantageous in wound healing and tissue engineering applications, where localized and timed therapeutic interventions are crucial.

### 2.2. Degradation-Independent Dynamic Hydrogels

Degradation-independent dynamic hydrogels, while robust, often encounter limitations in supporting prolonged 3D cell cultures, a vital component in numerous applications in tissue engineering and regenerative medicine [41]. The irreversible nature of their degradation can lead to inconsistencies in the network structure over time and across different spatial regions. This variability poses challenges in studies exploring the relationship between the local microenvironment and the behavior of encapsulated cells, as it can result in uneven cell growth and unpredictable tissue development [42].

**Figure 2:**
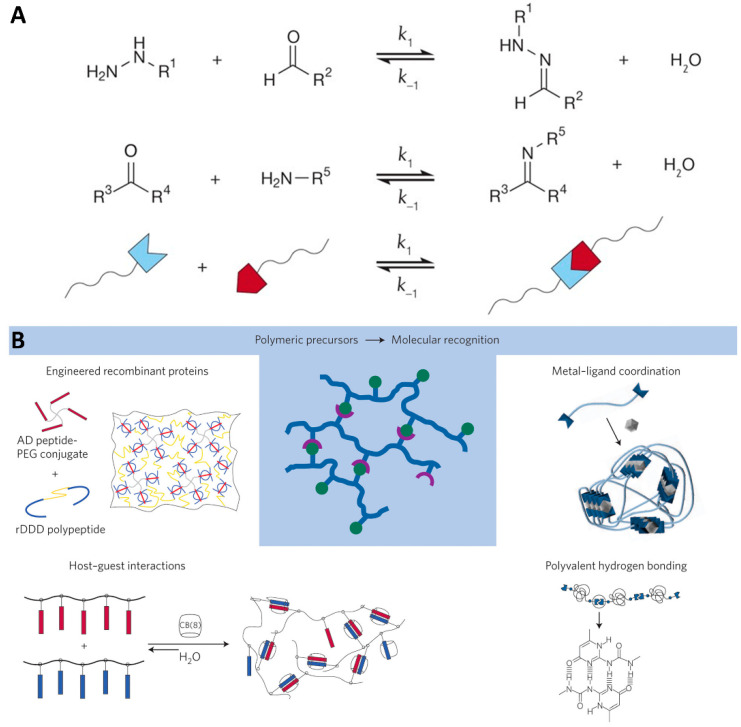
(**A**) Utilizing reversible covalent bonds to create degradation-independent dynamic hydrogels [67]. (**B**) Employing supramolecular recognition patterns in the design of degradation-independent dynamic hydrogels [68].

To emulate the dynamic characteristics of the natural ECM more accurately, researchers have developed dynamic hydrogels with reversible crosslinks that are not reliant on degradation. Generally, these reversible crosslinks in hydrogels can be categorized into two main types: reversible covalent bonds [43,44,45,46,47,48,49,50,51,52,53,54] (Figure 2A) and supramolecular physical connections [55,56,57,58,59,60,61,62,63,64,65,66]. The former encompasses a range of chemical interactions that can be reversed under certain conditions, providing a level of control and adaptability previously unattainable with traditional hydrogel systems. The latter encompasses metal–ligand coordination, host–guest complexation, and hydrogen bonding to create dynamic networks that respond to external stimuli and environmental changes (Figure 2B).

**Figure 3:**
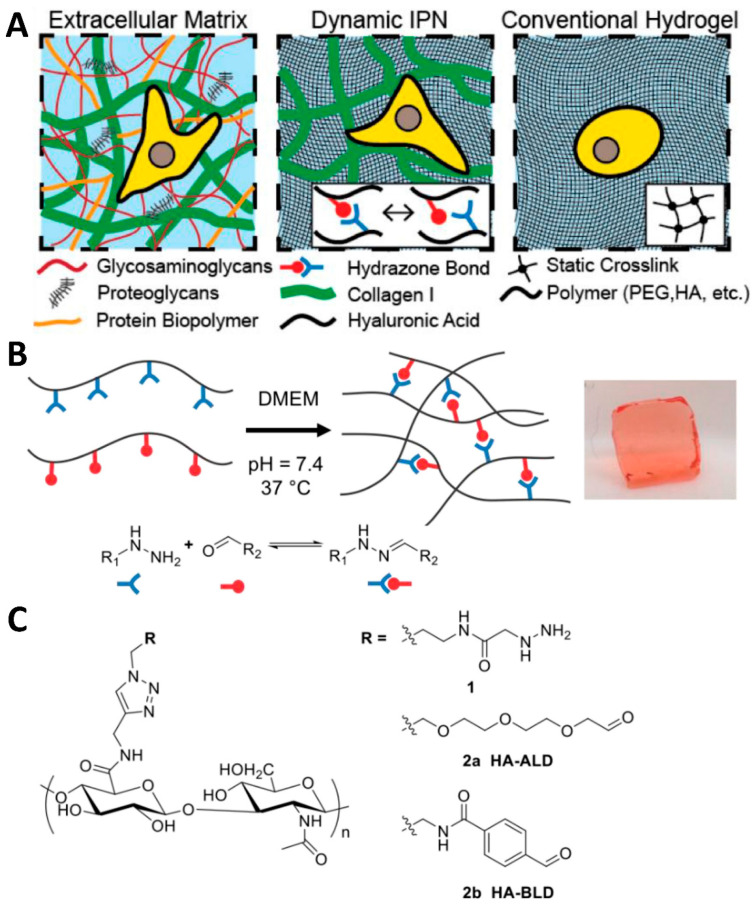
(**A**) Depiction of the architectures of the natural ECM dynamic HA-collagen. (**B**) Development of a dynamic hydrogel through reversible covalent hydrazone chemistry. (**C**) Molecular compositions of the modified HA [70].

The primary reversible reactions encompass Schiff base, boronate, and Diels–Alder reactions. Of these, Schiff base reactions, involving aldehyde and amine, hydrazide, or hydrazine groups, are particularly suited for biomedical applications due to their effective reversibility under physiological conditions. The introduction of catalysts in these reactions has expanded their potential, enabling more sophisticated control over the properties of hydrogels and interactions with embedded cells [69], which was demonstrated through the creation of a hydrogel platform based on hyaluronic acid (HA), incorporating catalysts, and anchored together by reversible covalent hydrazone bonds [70] (Figure 3A). The catalysts could be biocompatible, benzimidazole-based organocatalysts. These catalysts are specifically employed to enhance the formation and exchange of hydrazone bonds in hyaluronic acid-based hydrogels, thereby improving the injectability of hydrogels while maintaining their long-term stability. This innovation allows for more sophisticated control over the hydrogel properties and their interactions with embedded cells, addressing the challenge of balancing ease of injection with stability in cell transplantation and tissue engineering applications (Figure 3B).

**Figure 4:**
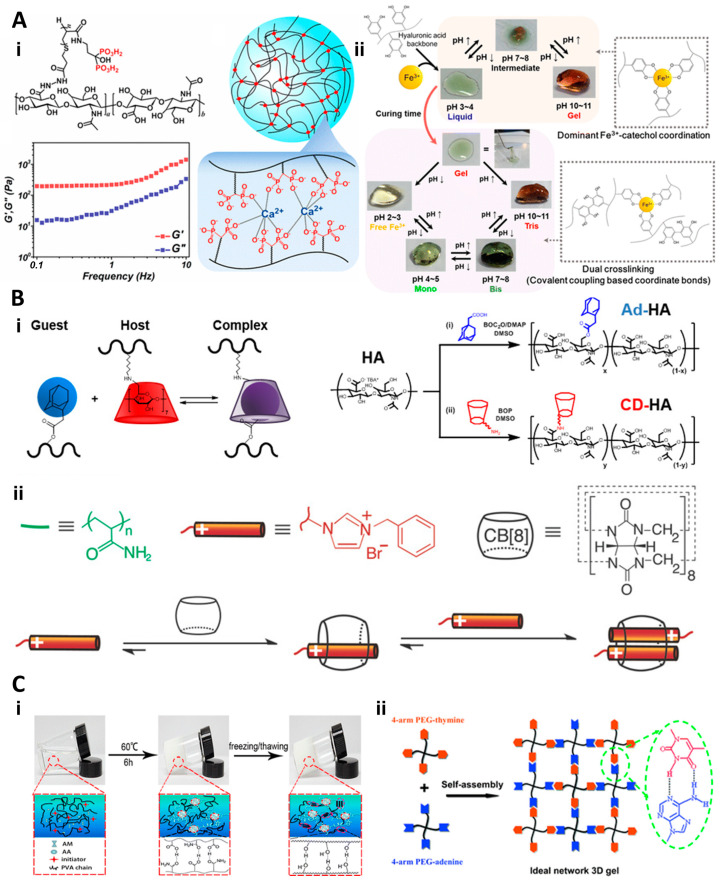
Hydrogels formulated through metal–ligand coordination for degradation-independent dynamics. (**A**) (**i**) A visual representation of a hydrogel structure created by HA-BP macromolecules, binding with Ca^2+^ ions [72]. (**ii**) A step-by-step depiction of HA-CA gel development across the pH spectrum, highlighting color transition [73]. (**B**) Hydrogels derived from metal–ligand coordination, ensuring degradation resistance. (**i**) A portrayal of the interaction between adamantane (Ad, guest) and β-cyclodextrin (CD, host) resulting in the formation of a reversible guest–host (GH) crosslink alongside the associated synthesis [75]. (**ii**) Sequential construction of a 2:1 ternary host–guest complex involving CB [8] and accompanying guest molecules [57]. (**C**) Hydrogels anchored by hydrogen bonds for persistent dynamic features. (**i**) A detailed diagram showcasing the formation of a completely physically crosslinked PVA/CP DN hydrogel [76]. (**ii**) An illustrative guide to the biological assembly of a four-arm PEG hydrogel lattice through hydrogen bonding of Watson–Crick base pair interactions between thymine and adenine structures [77].

The category of supramolecular physical crosslinks offers a different avenue for creating dynamic hydrogels. Metal–ligand coordination bonds, formed by the pairing of electrons between two linked atoms, are particularly interesting due to their unique response to external stimuli, making them ideal for creating stimuli-responsive hydrogels [71]. Ossipov and team introduced an injectable hydrogel that self-assembles from bisphosphonates and Ca^2+^ ions [72]. This hydrogel displays notable injectability during 3D bio-printing, with most encapsulated cells maintaining viability post-injection (Figure 4(Ai)). Sohn et al. outlined a hydrogel with phase adaptability, interconnected through reversible physical interactions, showing a pronounced phase transition in response to pH changes [73]. Host–guest complexation, another form of supramolecular interaction, involves the formation of complexes between two molecules, such as cyclodextrins (CDs) and synthetic cucurbit[n]urils (CBs) (Figure 4(Aii)) [74]. Burdick et al. designed a dynamic hydrogel anchored on “host-guest” interactions [75] (Figure 4(Bi)). Further studies indicated that this hydrogel can diminish inflammation in situ and enhance tissue regeneration. Beyond natural CDs, synthetic cucurbit[n]urils (CBs) have recently been employed for dynamic hydrogel creation. Scherman’s team produced a resilient and adhesive dynamic hydrogel, showcasing strong adhesion to various substrates [57] (Figure 4(Bii)). Hydrogen bonding represents another potent form of supramolecular interaction utilized in dynamic hydrogels. Wong’s group introduced a durable hydrogel anchored by hydrogen interactions. This hydrogel excels in dispersing the applied force [76] (Figure 4(Ci)). Additionally, Hu’s team presented a novel hydrogel composed of star-shaped PEG-based crosslinkers. This hydrogel can gradually release loaded drugs and direct behaviors of encapsulated cells [77] (Figure 4(Cii)). Together, these advancements in dynamic hydrogel technology represent a significant leap forward in the field of biomaterials. By offering a range of reversible crosslinking mechanisms, these hydrogels provide unparalleled versatility and adaptability, opening new horizons in tissue engineering, regenerative medicine, and drug delivery systems. Their ability to mimic the dynamics of natural ECM, respond to environmental stimuli, and support complex cellular interactions positions them as key materials in the development of next-generation biomedical solutions.

We summarize the different types of dynamic hydrogels, their unique properties (Table 1), and their specific applications in the field of biomedical engineering, offering a concise overview of the section.

### 2.3. The Advance of Engineered Dynamic Hydrogels

The advent of engineered dynamic hydrogels heralds a transformative era in the fields of biomedical engineering and regenerative medicine, especially in the context of treating degenerative endocrine diseases. These hydrogels, characterized by their responsive and adaptable nature, open up a myriad of possibilities for personalized medicine and advanced therapeutic strategies. This extended essay delves deeper into the facets of dynamic hydrogel technology, exploring its implications, challenges, and future prospects in greater detail.

#### 2.3.1. Revolutionizing Disease Management with Dynamic Hydrogels

Dynamic hydrogels have emerged as a beacon of hope in managing degenerative endocrine diseases, which are notoriously difficult to treat due to their complex nature and the need for precise and sustained intervention. These diseases, such as osteoporosis and diabetes, not only have a profound impact on individual health but also place a significant burden on healthcare systems globally. Dynamic hydrogels address these challenges by offering platforms for targeted drug delivery, hormone replacement therapies, and regenerative approaches that are finely tuned to the specific needs of each disease.

#### 2.3.2. Biophysical Regulation of Redox Homeostasis

At the heart of dynamic hydrogel technology is the ability to regulate redox homeostasis at the cellular level. Redox processes, involving the delicate balance between oxidation and reduction reactions in the body, are fundamental to maintaining cellular health. Disruptions in this balance are implicated in a range of pathological conditions, including degenerative endocrine diseases. Dynamic hydrogels, through their cell–material interactions, can modulate the redox environment, providing a therapeutic strategy that goes beyond symptom management to address the root causes of disease.

#### 2.3.3. Tailoring Hydrogels to Disease Specificities

The customization of dynamic hydrogels is a key aspect of their appeal. Depending on the disease, hydrogels can be engineered with specific properties, for instance, porosity, degradation rate, and mechanical strength, to match the requirements of the target tissue. In osteoporosis, hydrogels can be designed to enhance bone regeneration and mineralization, while in diabetes, they can be used for the controlled release of insulin and aid in wound healing. This level of customization ensures that the treatment is not only effective but also minimizes potential side effects.

#### 2.3.4. The Role in Stem Cell Therapy and Regenerative Medicine

Dynamic hydrogels are particularly promising in the realms of stem cell therapy and tissue engineering. Their ability to mimic the natural extracellular matrix creates an ideal environment for stem cells to proliferate and differentiate. By manipulating the mechanical and chemical properties of the hydrogel, it is possible to guide stem cells towards specific lineages, enhancing the efficacy of regenerative treatments. This aspect is crucial in developing therapies for diseases where tissue regeneration is a primary goal, such as in the restoration of pancreatic beta cells in diabetes or bone tissue in osteoporosis.

## 3. The Metabolism Regulation of Dynamic Hydrogels

### 3.1. Osteoporosis

**Figure 5:**
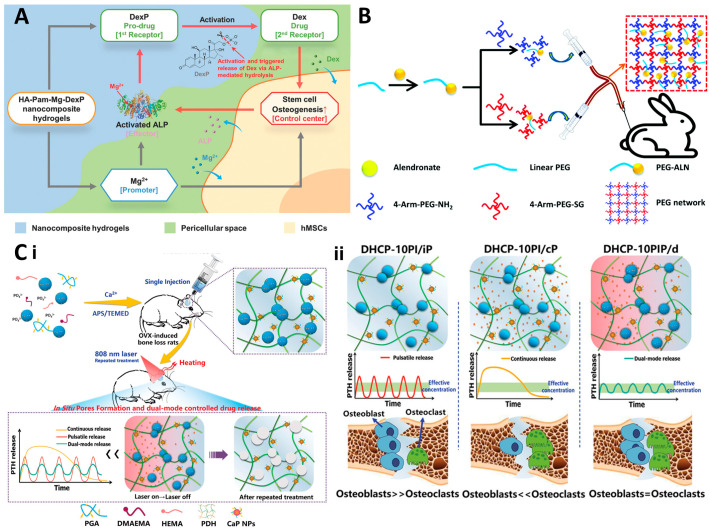
(**A**) The schematic positive feedback of ALP-mediated biomineralization in bisphosphonate-functionalized hydrogel. (**B**) The design of alendronate-loaded tetra-PEG hydrogel. (**C**) PTH-loaded injectable hydrogel. (**i**) The schematic hydrogel network and the mechanism of NIR-controlled pulsatile PTH release. (**ii**) The schematic PTH release-mediated manipulation of the redox balance of osteoblasts and osteoclasts.

Osteoporosis is a typical hormone-level-related degenerative disease. A proper level of hormones, such as estrogen and testosterone, is essential for the maintenance of bone mineral density (BMD) [78]. The disease is notably prevalent among postmenopausal women, where a significant reduction in estrogen levels leads to a decrease in BMD, elevating the risk of fractures [79,80]. This condition underscores a critical aspect of bone health, where hormonal balance plays a pivotal role in maintaining bone strength and integrity. To combat the onset and progression of osteoporosis, hormone replacement therapy (HRT) was traditionally employed, aiming to replenish the declining hormone levels and thereby reduce bone density loss [81,82,83]. However, while effective in preserving bone mass, HRT has been associated with increased risks of cardiovascular events, such as heart attacks and strokes, posing a dilemma for patients with existing heart and brain health concerns [83]. Recently, bisphosphonates have been widely used to treat osteoporosis in postmenopausal women with high heart and brain disease risks [84]. Bisphosphonates, known for their bone-preserving properties, act by inhibiting osteoclast-mediated bone resorption, thereby slowing down the process of bone loss. Their incorporation into dynamic hydrogel systems represents an innovative approach, combining the therapeutic efficacy of bisphosphonates with the versatile delivery capabilities of hydrogels. Zhang and coworkers reported a novel bisphosphonate-functionalized hyaluronate-based system [85,86,87,88]. This hydrogel has demonstrated significant potential in enhancing osteogenesis in mesenchymal stem cells (MSCs) and facilitating effective bone regeneration. It achieves this through the redox regulation of alkaline phosphatase (ALP)-mediated biomineralization, a process crucial for bone formation [86] (Figure 5A). Nafee et al. developed an alendronate-loaded and biodegradable smart hydrogel [89]. The reported hydrogel shows excellent biocompatibility and biodegradation in vivo. Moreover, it has a high affinity for the hard tissues and promotes their regeneration via the redox regulation of bone homeostasis. Li et al. reported a tetra-PEG hydrogel to achieve long-term alendronate delivery [90]. The design of the on-demand release of alendronate provides a smart platform to optimize the bone regeneration of rabbits with osteoporosis (Figure 5B). Dynamic hydrogels loaded with hormones or hormone replacements are also attractive candidates for osteoporosis therapy. Kuang et al. designed an injectable hydrogel capable of near-infrared (NIR) light-controlled pulsatile parathyroid hormone (PTH) release [91] (Figure 5(Ci)). The pulsatile PTH release can be further manipulated to finely tune the redox balance of osteoblasts and osteoclasts, thereby altering bone homeostasis to promote bone regeneration in the osteoporosis model (Figure 5(Cii)). Amani et al. [92] and Chen et al. [93] reported two hydrogels loaded with teriparatide, an anabolic drug for osteoporosis treatment. These hydrogels offer a new paradigm in drug delivery, potentially enhancing the drug’s therapeutic impact while simultaneously reducing the side effects associated with its systemic administration. The integration of dynamic hydrogels in osteoporosis treatment heralds a new era in personalized medicine. By offering controlled, targeted, and sustained delivery of therapeutic agents, these hydrogels not only improve treatment efficacy but also significantly reduce the potential risks associated with traditional osteoporosis therapies.

### 3.2. Type II Diabetes

**Figure 6:**
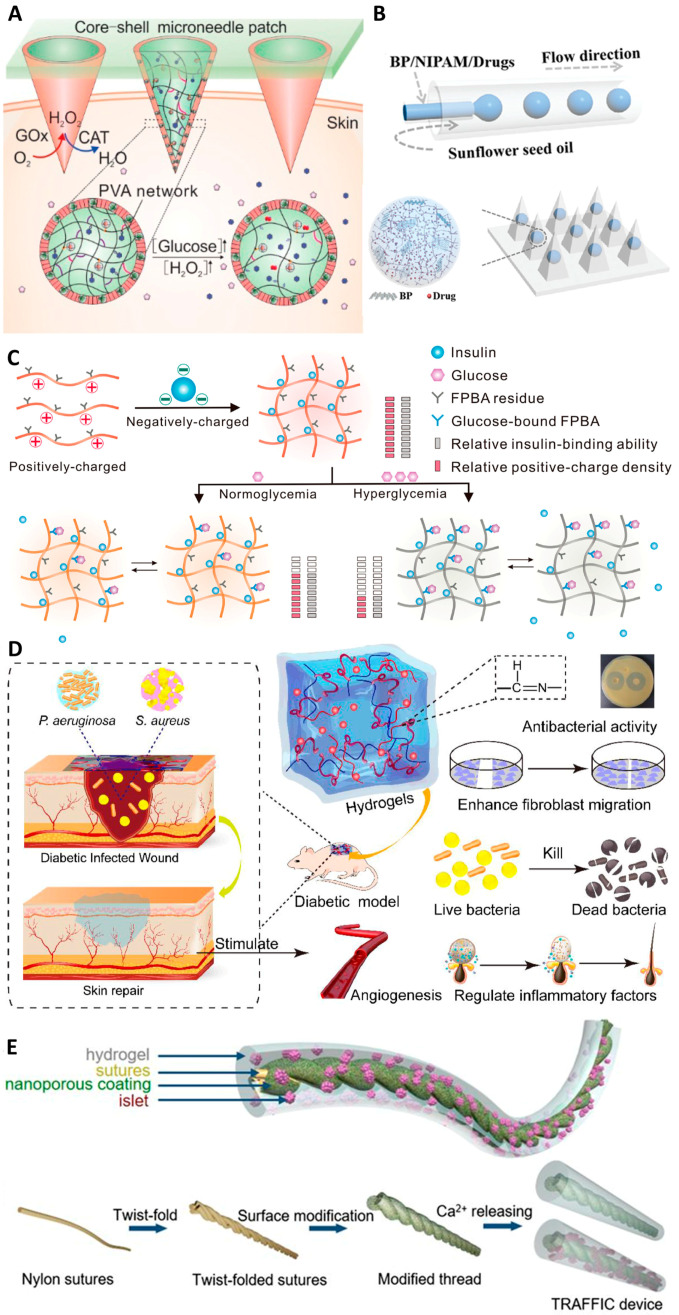
(**A**) The schematic illustration of bio-responsive insulin-delivery microneedle system. (**B**) The design of responsive hydrogel microcarrier-integrated microneedles. (**C**) The schematic illustration of electrostatic interaction-driven insulin release. (**D**) The schematic illustration of dynamic hydrogels for wound healing in diabetes. (**E**) The design of cell encapsulation device for the reconstruction of pancreatic function.

Type II diabetes is a chronic condition predominantly caused by pancreatic β-cell dysfunction and insulin resistance. It is characterized by an imbalance in redox homeostasis, leading to elevated blood glucose levels [94]. Early intervention and management of blood glucose are crucial in preventing the progression of the disease and averting a myriad of complications, including cardiovascular diseases, neuropathy, nephropathy, and retinopathy [95,96,97]. In past studies, great advances have been made in using dynamic hydrogels to treat type II diabetes through insulin delivery, wound healing, and stem cell therapy [98]. Wang et al. designed a core-shell microneedle patch based on dynamic PVA hydrogel [99]. This bioinspired system, coated with hydrogen peroxide (H_2_O_2_)-scavenging enzymes, mimics the function of peroxisomes, enabling site-specific insulin delivery. This method significantly minimizes off-target effects and toxicities associated with conventional insulin therapies, offering a more precise and controlled treatment modality (Figure 6A). Fan et al. reported a photo-responsive insulin delivery to realize on-demand control of blood glucose concentration [100] (Figure 6B). This innovation enables on-demand control of blood glucose concentration through light-triggered release mechanisms, providing patients with a more flexible and responsive way to manage their diabetes. Wang et al. reported an injectable dynamic polymeric complex for glucose-responsive insulin delivery [101]. The binding affinity between the polymeric complex and insulin can be changed in response to the blood glucose level (Figure 6C). In hyperglycemia, the insulin release is smartly triggered to achieve real-time and effective blood glucose regulation. Xiao et al. reported a metal–organic framework (MOF) for enhanced skin protection and regeneration in diabetes [102]. The developed hydrogel system, through redox antioxidant regulation, significantly promotes angiogenesis in wound healing, addressing one of the common complications of diabetes. Zhu et al. designed an antioxidant dynamic hydrogel loaded with stromal cell-derived factor-1 (SDF-1) (Figure 6D). The sustained release of SDF-1 from this hydrogel enhances angiogenesis and re-epithelialization in diabetic wounds, aiding in faster and more effective healing. This process is facilitated by the redox regulation of keratinocyte differentiation, which is crucial for skin repair [103]. Wei et al. reported a dynamic hydrogel based on Schiff’s base reaction capable of the delivery of antimicrobial peptides for wound healing [104]. The developed hydrogels can expedite wound healing through the redox regulation of the inflammatory response and collagen deposition. Dynamic hydrogels are also excellent vehicles for the delivery of therapeutic stem cells for the reconstruction of pancreatic function. An et al. reported a cell encapsulation device for the replacement of pancreatic β-cells with dysfunctionality [105] (Figure 6E). The developed device can be further modified and used for other endocrine disorders and hormone-deficient diseases. The application of dynamic hydrogels in managing type II diabetes represents a significant leap in diabetes care. The integration of dynamic hydrogels in diabetes treatment is a testament to the advancements in biomedical engineering and material science. These hydrogels not only offer more effective and patient-friendly options for managing diabetes but also hold the promise of addressing the underlying causes of the disease.

### 3.3. Other Degenerative Endocrine Diseases

**Figure 7:**
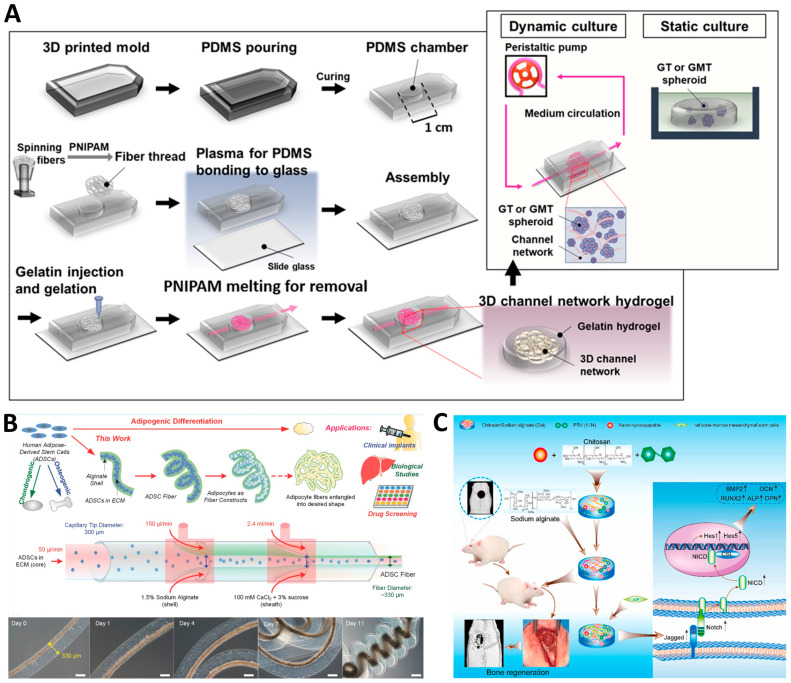
(**A**) The design of ovary spheroid culture in a 3D channel network hydrogel. (**B**) The design of microfiber hydrogel for ADSC encapsulation. (**C**) The design of hydrogel for PTH delivery to promote bone regeneration.

Dynamic hydrogels are proving to be a transformative tool in the treatment of a range of degenerative endocrine diseases, offering innovative methods for hormone delivery, drug release, and the regulation of endocrine functions. These hydrogels provide a platform for sustained, controlled, and targeted therapy, essential in managing diseases characterized by hormonal imbalances and metabolic dysregulation. There are many pilot studies giving the proof-of-concept results of these platforms for various endocrine and redox disorders, including hyperlipidemia, ovary dysfunction, obesity, and hypoparathyroidism. Sirc et al. reported a poly (2-hydroxyethyl methacrylate) (PHEMA) and poly (N-vinylpyrrolidone) (PVP) copolymer-based hydrogel for the delivery of niacin to reduce the blood fat in hyperlipidemia [106]. The sustained release mechanism of hydrogels ensures that niacin is delivered over an extended period, maintaining blood lipid levels within a healthy range and potentially reducing the risk of cardiovascular complications associated with hyperlipidemia. Yoon et al. designed a vascularized hydrogel for the delivery of ovary spheroids [107]. By delivering ovary spheroids, this hydrogel system supports hormone autocrine functions, which could be pivotal in restoring ovarian health and hormonal balance, offering hope for those suffering from conditions such as polycystic ovary syndrome (PCOS) and early menopause. (Figure 7A). Hsiao et al. developed an adipose-derived stem cell (ADSC) fiber-based hydrogel for the 3D encapsulation of unilocular adipocytes [108]. Adipose tissue is an important organ with the ability to regulate various endocrine and redox processes. This microfiber hydrogel can be used to fabricate the device for the early diagnosis and treatment of obesity-related diseases (Figure 7B). Zou et al. reported a hydrogel for the delivery of parathyroid hormone (PTH) [109]. By regulating the redox balance between osteoblasts and osteoclasts, this hydrogel promotes bone regeneration, addressing the key issue of bone loss in degenerative bone diseases (Figure 7C). Park et al. reported a gelatin hydrogel for the sustained release of PTH to rescue the therapeutic potential of tonsil-derived mesenchymal stem cells (TDMSCs) for hypoparathyroidism [110]. Hypoparathyroidism, a condition marked by the insufficient production of parathyroid hormone, can lead to a range of complications, and this innovative approach offers a new avenue for treatment. The use of dynamic hydrogels in managing degenerative endocrine diseases represents a significant advancement in medical science. These hydrogels offer customized treatment options that can be tailored to the specific needs of individual patients, providing sustained and controlled delivery of therapeutic agents.

## 4. Summary and Perspective

Engineered dynamic hydrogels are excellent platforms to employ cell–material interactions to biophysically regulate redox homeostasis, thereby rescuing many degenerative endocrine diseases. Moreover, they can be finely tailored for the delivery of hormones, hormone replacements, and therapeutic drugs according to the unique characteristics of degenerative endocrine diseases. In this review, we summarize the recent state-of-the-art developments of dynamic hydrogels and review the biophysical manipulation of cell fates through the rationally designed hydrogel network. Finally, we also summarize the biomedical applications of dynamic hydrogels in degenerative endocrine diseases. By shedding light on the engineered dynamic hydrogel niches, we would like to enhance the tools for the manipulation of cell behaviors and to guide biomaterials’ design and their biomedical applications.

However, the journey of dynamic hydrogels from laboratory research to clinical application in the fields of regenerative medicine and biomedical engineering is a path laden with potential yet fraught with challenges. These hydrogels, promising in treating degenerative endocrine diseases, face a multi-faceted challenge in their transition to practical, clinical use.

The complexity of moving from controlled laboratory experiments to unpredictable clinical environments is substantial. Laboratory conditions are ideal and precisely controlled, but clinical settings present variables such as differing patient responses, complex disease states, and practical limitations in medical settings. Ensuring that dynamic hydrogels perform as expected in real-world scenarios is a significant hurdle.

The scalability of producing dynamic hydrogels is another critical issue. While producing these hydrogels in small quantities for research purposes is feasible, mass-producing them for widespread clinical use is a different challenge. It involves not only manufacturing challenges but also logistical, regulatory, and cost considerations. Ensuring consistent quality and performance at a larger scale is essential.

The safety and efficacy of dynamic hydrogels in humans are paramount. Long-term studies and rigorous clinical trials are required to establish their safety profile and effectiveness in treating specific diseases. This process is time-consuming and costly but essential to gain regulatory approval and medical acceptance.

The integration of dynamic hydrogels with cutting-edge technologies like nanotechnology and 3D bioprinting presents both opportunities and challenges. These integrations could enhance the capabilities of hydrogels, offering more precise and personalized treatment options. However, this also adds layers of complexity in terms of design, production, and testing. Ensuring compatibility and optimizing these integrated systems for clinical use are crucial steps.

## Figures and Tables

**Table 1:** Summary of degradation-reliant and degradation-independent dynamic hydrogels.

Type	Sub-Type	Key Features	Mechanism of Action	Specific Examples and References	Biomedical Applications
Degradation-reliant dynamic hydrogels	Hydrolytic	Maintain integrity, allow controlled degradation	Hydrolysis of covalent interactions, tunable degradation rate	HEMA-modified polysaccharide hydrogels [37], PEG-oriented hydrogels [38]	Drug delivery, controlled exposure of embedded cells
	Enzymatic	Feature enzyme-cleavable peptide sequences	Remodeling via cell-mediated enzymatic degradation	MMP-sensitive HA hydrogels [39], enzyme-responsive protein structures [23]	Replicating natural tissue dynamics, supporting cell adhesion and proliferation
	Light responsive	Degrade upon specific light exposure	Light-induced breakdown, spatial/temporal control	Nitrobenzyl-crosslinked hydrogels [39,40]	Wound healing, localized therapeutic interventions
Degradation-independent dynamic hydrogels	Reversible covalent	Reversible bonds under certain conditions	Schiff base, boronate, Diels–Alder reactions	Hydrazone-bonded HA hydrogels [70]	Injectable platforms, cell therapy
	Supramolecular physical	Formed by non-covalent interactions	Metal–ligand coordination, host–guest complexation, hydrogen bonding	Metal–ligand coordinated hydrogels [72], guest–host interaction hydrogels [75]	Stimuli-responsive hydrogels, phase adaptability in response to environmental changes

## Data Availability

Not applicable.
